# Quantification of redox thermodynamics shifts within coacervates

**DOI:** 10.1073/pnas.2521526122

**Published:** 2025-11-14

**Authors:** Gala Rodriguez, Nicholas B. Watkins, Xagros Faraji, Elizabeth Lee, Lior Sepunaru

**Affiliations:** ^a^Department of Chemistry and Biochemistry, University of California at Santa Barbara, Santa Barbara, CA 93106

**Keywords:** coacervates, temperature dependent electrochemistry, origin of life, reaction thermodynamics, microenvironment

## Abstract

Coacervates are suggested to be viable protoenzymes due to their propensity to act as catalytic microreactors for biochemical reactions. However, the mechanism by which they alter reaction thermodynamics remains unclear. While extensive research has been conducted displaying the ability of coacervates to compartmentalize a wide variety of reactants, products, and catalysts, insight into how reactant, transition state, and product energies are altered within the droplet continues to be an active area of research. One promising strategy for investigating the thermodynamics and kinetics within the coacervate phase is temperature-dependent electrochemistry, which enables the extraction of reaction entropy, enthalpy, and Gibbs energy. In this work, we use ferri/ferrocyanide, a well-behaved redox couple that has been proposed to be an essential oxidizing agent in prebiotic Earth, to investigate the microenvironment created by the coacervation of poly-L-lysine and polyuridylic acid. We observe an oxidative shift upon partitioning into the coacervates, which temperature-dependent experiments reveal is due to a 40 J/mol K and an 8 kJ/mol increase in reaction entropy and enthalpy, respectively. We attribute the change in entropy to a highly structured water hydrogen-bonding network within the droplets and, subsequently, around the redox probe. Further, we reveal via in situ Raman measurements that the change in reaction enthalpy is due to the destabilization of the product, ferrocyanide, within the ionic coacervate phase.

## Significance

The earliest enzymes are thought to have formed through the assembly of macromolecules into disordered, secondary phases known as coacervates. While these phases are believed to have played a role in early catalysis, the underlying mechanisms remain poorly understood. Here, we use temperature-dependent electrochemistry to investigate how confinement within coacervates and the resulting increase in local charge concentration affect the reduction of ferricyanide to ferrocyanide. Our results show a decrease in reaction entropy within the coacervate environment, and Raman spectroscopy reveals an inverse relationship in stabilization energy between the reactant and product states. Together, we provide an analytical quantification of changes in reaction thermodynamics within coacervates and offer insights into the chemistry of early life.

Electron transfer (ET) is a pillar for both mammalian and bacterial metabolism, with oxidoreductases constituting roughly a third of all classified enzymes (1). In biological systems, enzymes have evolved over millions of years to become hyperselective micromachines, hosting specific interactions between the reactant, cofactor, and enzyme to direct reactivity and to accelerate the ET reaction by lowering the transition state energy (2, 3). Mechanistic studies have shown that minimization of the reaction entropy is dominant for reactions with multiple substrates or with large solvent reorganization energies, while most enthalpic contributions come from active site preorganization and bringing reactants to a near-attack conformation. (2, 4) As a result of these interactions, enzymes are capable of accelerating reaction kinetics by orders of magnitude (5, 6). It has been proposed that promiscuous enzymes are model systems for protoenzymes, as they have low substrate specificity (7–9). This range in conformational ensembles is shared between intrinsically disordered proteins as well as enzymes; the former has a high propensity to undergo liquid–liquid phase separation (LLPS) (10).

Coacervates, one type of LLPS, are speculated to be the first biological catalysts that arose from the primordial soup according to the Oparin–Haldane hypothesis (11). These secondary phases are formed by the association of polymers via inter- and intramolecular interactions, but their lack of structural specificity precludes the formation of well-defined boundaries, like a membrane. However, because it is a distinct secondary phase, intermolecular interactions can drive the partitioning of species into or out of the coacervate droplets (12). The ability of coacervates to drive molecular motion, along with their potential to grow or dissipate in response to environmental changes such as in ionic strength or pH, make them stimuli-responsive materials capable of supporting a wide range of biochemical reactions (13). Notably, the most common form of coacervates in biological systems is membraneless organelles (MLOs), or LLPS between proteins, mRNA, and a slew of molecules (14–16). Thus far, the predominant mechanisms by which coacervates are known to facilitate biological function are the concentration of species via partitioning to accelerate reaction kinetics; the exclusion of water or protons to change the stability of reactants, transition states, or products; and the generation of an electric potential gradient at the liquid–liquid interface to facilitate redox reactions (17–21). For example, recent work with coacervates composed of polylysine and Fe(CN)_6_^3−^ showed significantly accelerated amide bond formation between acetylated α-amidothioacids and amino acids. While this reaction can be accomplished in the absence of LLPS, reactivity is extremely slow due to low bulk Fe(CN)_6_^3−^ concentrations. The formation of LLPS creates a high local concentration of ferricyanide, which enables rapid amide bond formation upon the partitioning of reactants. In addition, the work investigates other model systems composed of polyarginine and Fe(III) that facilitate the oxidation of both NADPH and glutathione substrates effectively highlighting coacervate’s catalytic promiscuity (22). With other systems, ratiometric pH-sensitive dyes have been used to monitor the pH within the coacervate phase and reveal changes in pH by up to 3 pH units (23). In the case of interfacial electric fields, the surface potential of biomolecular condensates can extend up to a 100 mV difference, enabling reactions such as the oxidation of hydroxide to hydrogen peroxide (20, 21). However, explicit identification of the specific interactions that trigger changes in reactant and product energies within the secondary coacervate phase remains elusive.

[1] $$\Delta G=-nFE_{f}.$$

**Fig. 1.**
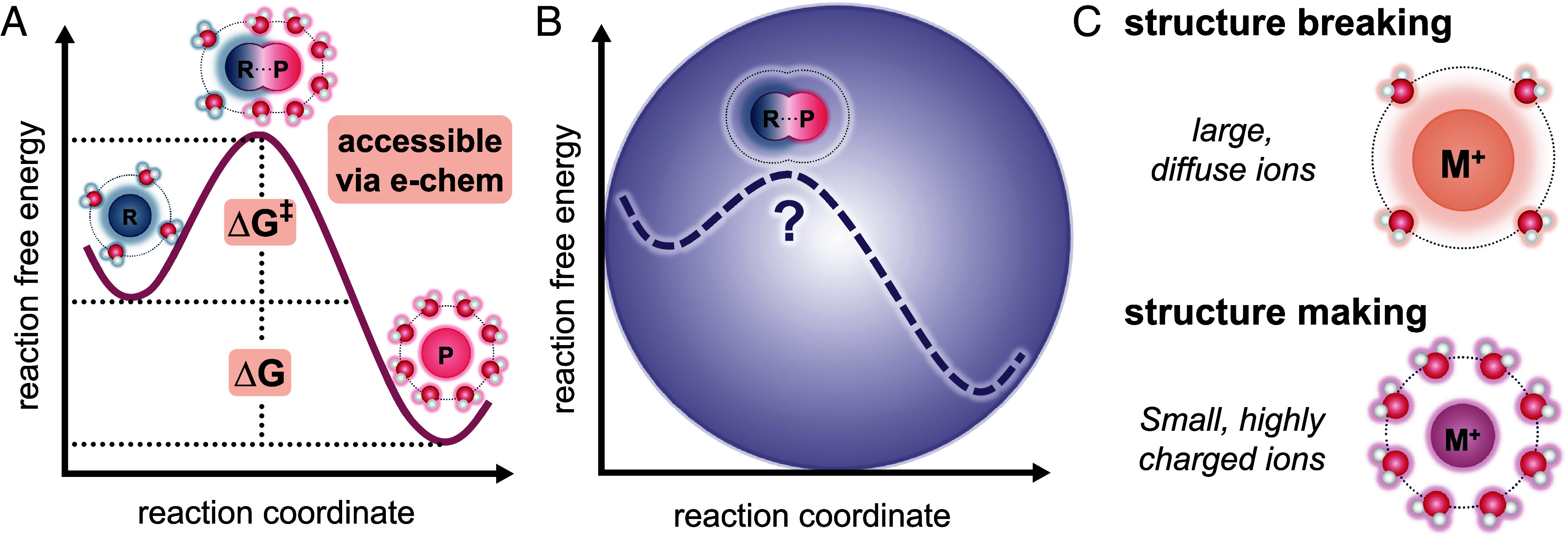
Electrochemistry provides insight into the thermodynamic driving forces that drive chemical reactions. (*A*) As reactants (R) are converted to products, it surmounts a potential energy barrier (ΔG^‡^) in order to form the product (P). The difference between the product and reactant is otherwise known as the Gibbs energy (ΔG). Both ΔG^‡^ and ΔG are accessible utilizing electrochemistry (orange). (*B*) Once confined within the coacervate, the reaction pathway deviates according to the internal coacervate microenvironment. (*C*) The microenvironment is largely dictated by the surrounding water and ion reorganization and whether they induce or break local hydrogen-bonding networks.

Such analysis has been performed on biphasic systems, such as water droplets in oil, to reveal the influence of counterions and confinement on reactivity (24–26). Accordingly, we believed that electrochemistry could be used to elucidate how confinement within coacervates alters reaction thermodynamics. Coacervates commonly exclude a significant amount of water from their internal environment, which is known to affect the energies of the reactant, transition state, and product states (Fig. 1*B*) (19, 27, 28). A similar effect can be observed with ion-induced changes to reaction thermodynamics with the ferri/ferrocyanide redox reaction across a series of alkali metal counter cations using electrochemical methods (29, 30). Ions with small radii, such as lithium, act like point charges and create highly structured water networks, whereas ions with larger radii, like cesium, have more diffuse charge and disorder the local water structure (Fig. 1*C*). Recent work from the group of Yang Shao-Horn shows clear cation-dependent trends associated with solvent structure and reaction thermodynamics for ferri/ferrocyanide (*SI Appendix*, Table S1) (29). Increasing the cation radius from Li^+^ to Cs^+^ causes the system to become more exergonic, exothermic, and structured. A more negative ∆H is consistent with the weaker hydrogen bonding networks (HBN) associated with Cs^+^ enabling more direct, stabilizing interactions of the cations with the ferrocyanide. Since ferrocyanide is known to bind water molecules more strongly than ferricyanide, we expect that changes in its solvation shell would be the main driver for the changes in the reaction coordinate diagram (31, 32). It is worth noting that beyond ionic composition, ionic concentration is also strongly tied to the thermodynamics and kinetics of the system, as indicated by ion concentration-dependent formal potential and exchange current values (29, 33, 34).

In this study, we extract thermodynamic parameters associated with the ferri/ferrocyanide model reaction inside coacervate droplets utilizing temperature-dependent electrochemistry. By relating Eq. **1** to the Gibbs energy (Eq. **2**) and taking a temperature-dependent derivative, we generate Eq. **3**, which relates the ∆S of the reaction to the temperature-dependent formal potential of the reaction, E_f_(T). Further, since ∆G is directly measured using voltammetry and Eq. **1**, we can plug the extracted value for ∆S into Eq. **2** to create a self-consistent value for ∆H (35). We note that these relationships are only valid based on the assumption that the reaction entropy and enthalpy are not temperature-dependent terms at constant pressure within the small temperature range tested (20 to 40 °C) (36).

[2] ΔG=ΔH-TΔS,

[3] $$\delta E_{f}(T)/\delta T=\Delta S/nF.$$

## Results and Discussion

**Fig. 2.**
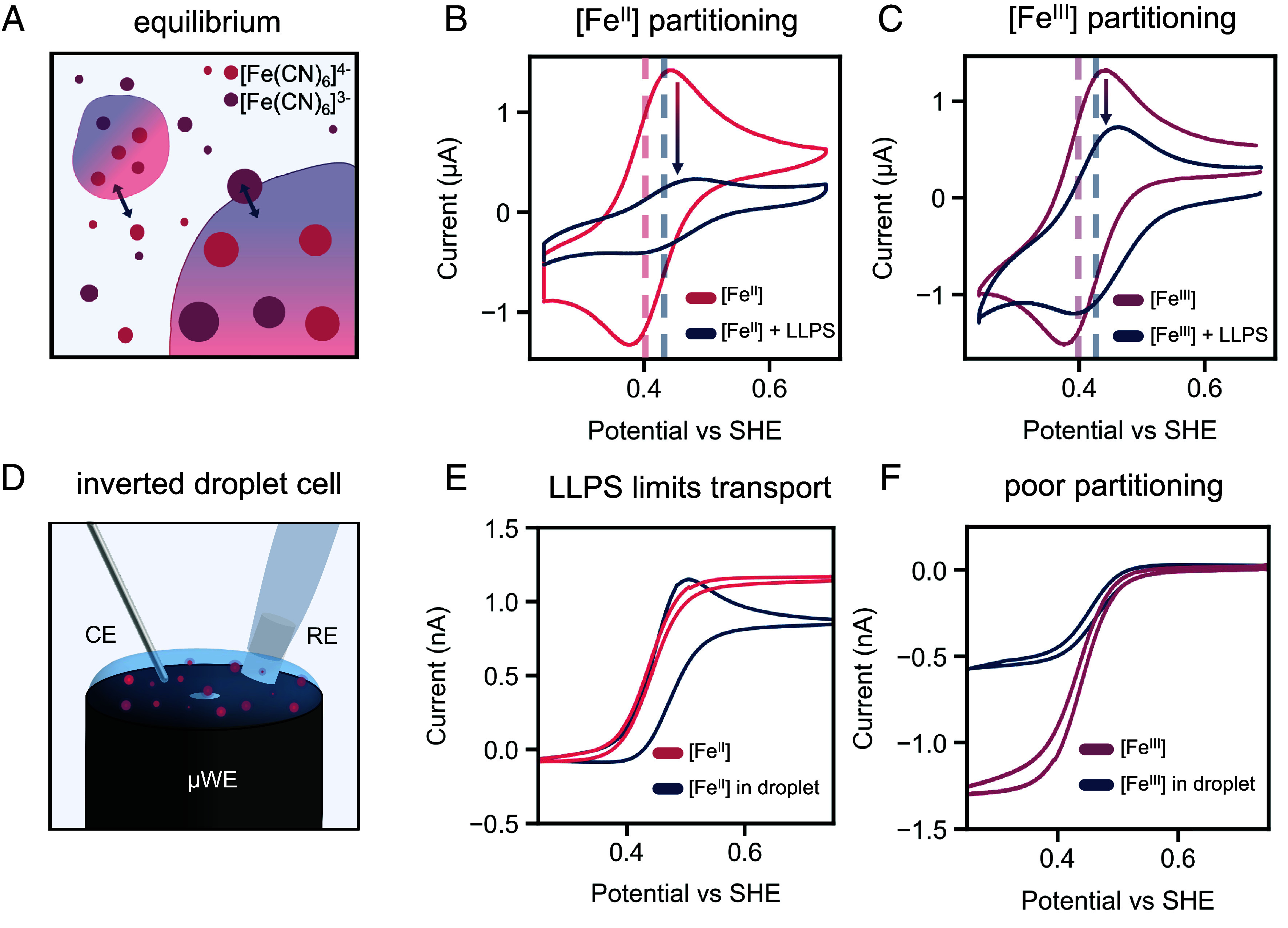
Electrochemically quantifying the effect of species charge on partitioning. (*A*) Schematic of the partitioning of ferrocyanide [pink, Fe(CN)_6_^4–^)] and ferricyanide [purple, Fe(CN)_6_^3–^] into coacervate droplets. Macroelectrode (3 mm diameter) cyclic voltammograms of 0.5 mM (*B*) ferrocyanide and (*C*) ferricyanide with and without LLPS made from 1 mg/mL of polyK_50_ and polyU. Peak current attenuation is indicative of ferrocyanide partitioning as denoted from the arrows. The dashed lines denote the 20 mV shift in Fe(II) and Fe(III) formal potential with (pink/purple) and without (blue) coacervates present in solution. (*D*) Schematic of a 10 µm Pt microelectrode inverted droplet cell. Microelectrode CVs of a droplet containing 1 mM (*E*) ferrocyanide (pink) and (*F*) ferrocyanide (purple) in an LLPS solution containing 1 mg/mL polyK_50_ and polyU (blue). We display the LLPS CVs after 20 cycles, once the droplets in solution have had time to land onto the microelectrode. All CVs were performed in 40 mM KCl with a platinum working electrode and a 20 mV/s scan rate.

Despite the propensity for polyK to coacervate with small and highly charged ferri/ferrocyanide ions, we chose a combination of a 50-mer poly-L-lysine (polyK_50_) with polyurydilic acid (polyU) at a roughly 2:1 cation to anion charge ratio as a model coacervate system (22). Unlike the former coacervate system, this combination maintains its partitioning affinity toward ferri/ferrocyanide ions into polycationic peptide-based coacervates while remaining stable in the absence of iron species (*SI Appendix*, Fig. S1*C*). In addition, this polyelectrolyte combination creates larger droplets, a feature that increases the probability of direct electrochemical sensing. Spherical droplets ca. 0.5 to 3 µm in diameter were obtained both in the presence and absence of 1 mM ferri/ferrocyanide in 40 mM KCl (*SI Appendix*, Figs. S1–S3). Although droplets slowly grew in size at 20 ºC, at higher temperatures they remained constant for 30 min at relevant electrolyte concentrations (40 mM KCl). Suggesting past 20 min, coacervates coalescence across all temperatures, with negligible changes in size before then. Therefore, initial experiments were performed within a 15-minute time frame to compare similar-sized coacervates across all temperatures (*SI Appendix*, Figs. S4 and S5). It is worth noting that while we used 40 mM KCl, the total electrolyte concentration, including polymer counterions (~3 mM from polyU·Na^+^ and ~6 mM from polyK_50_·HCl), is approximately 90 mM with a total estimated ionic strength of 45 mM. Initial electrochemical measurements with ferri/ferrocyanide and the LLPS system showed a significant decrease in cyclic voltammogram intensity (Fig. 2 *B* and *C*). We attributed this decrease in electrochemical signal to the partitioning of the redox ion into the coacervate. Once inside the droplet, the redox molecule is no longer electrochemically active, suggesting the coacervate is insulating. The redox couple remaining in solution exhibits a 20 mV shift in formal potential relative to the free complex, which we associate with coordination to trace amounts of unintegrated polyK_50_ in solution (*SI Appendix*, Fig. S6). This suggests that polyK_50_ is not only acting as a coacervate component but is actively participating as a chelating agent—unlike polyU which exhibits no electrostatic effect on the redox species formal potential. We expect the increase in charge associated with ferrocyanide compared to ferricyanide to be the reason for higher partitioning. To investigate the electrochemistry within the coacervate droplets, we used an inverted microelectrode droplet cell to allow gravity to bring droplets to the electrode surface and for the impact of droplets to immediately affect the current observed (Fig. 2*D* and *SI Appendix*, Fig. S7) (37). Interestingly, when ferrocyanide coacervates are left in solution and measured over the course of 20 cycles, a peak associated with mass transport limitations appears, suggesting the coacervates are crashing out of solution and forming a film (Fig. 2*E* and *SI Appendix*, Fig. S8*A*). Typically, microelectrodes produce CVs with flat steady-state regions due to constant radial flux to the electrode surface, as seen in the ferrocyanide control. However, film formation establishes a new steady state after 20 cycles, as the redox probe must first diffuse through the film before ET, introducing mass transport limitations and producing a CV reminiscent of a macroelectrode. The absence of a peak on the reductive sweep is then likely due to ferricyanide’s low partition coefficient within coacervates and films. This is confirmed when this experiment is performed with ferricyanide in the coacervate solution. Over the course of twenty cycles, there is a 50% loss in ferricyanide peak current consistent with a low partition coefficient into the coacervate phase (Fig. 2*F* and *SI Appendix*, Fig. S8*B*). By performing CVs at faster scan rates, we were able to follow the shifting of the formal potential of the iron complex over time, with the E_f_ plateauing around 0.460 V vs SHE, a roughly 35 mV shift (*SI Appendix*, Fig. S9). All experiments were performed within a potential window of –0.041 to +0.84 V vs SHE, which is well below the onset of pyridine oxidation (+1.797 V) (38). Furthermore, platinum electrodes were chosen specifically to minimize the formation of reactive oxygen species, since oxygen is reduced predominantly to water on Pt, and to avoid electrode fouling by the ferri/ferrocyanide couple (39). Together, these results support a change in the reaction thermodynamics within the coacervate phase, but indicate a more direct approach is required to quantify this shift.

**Fig. 3.**
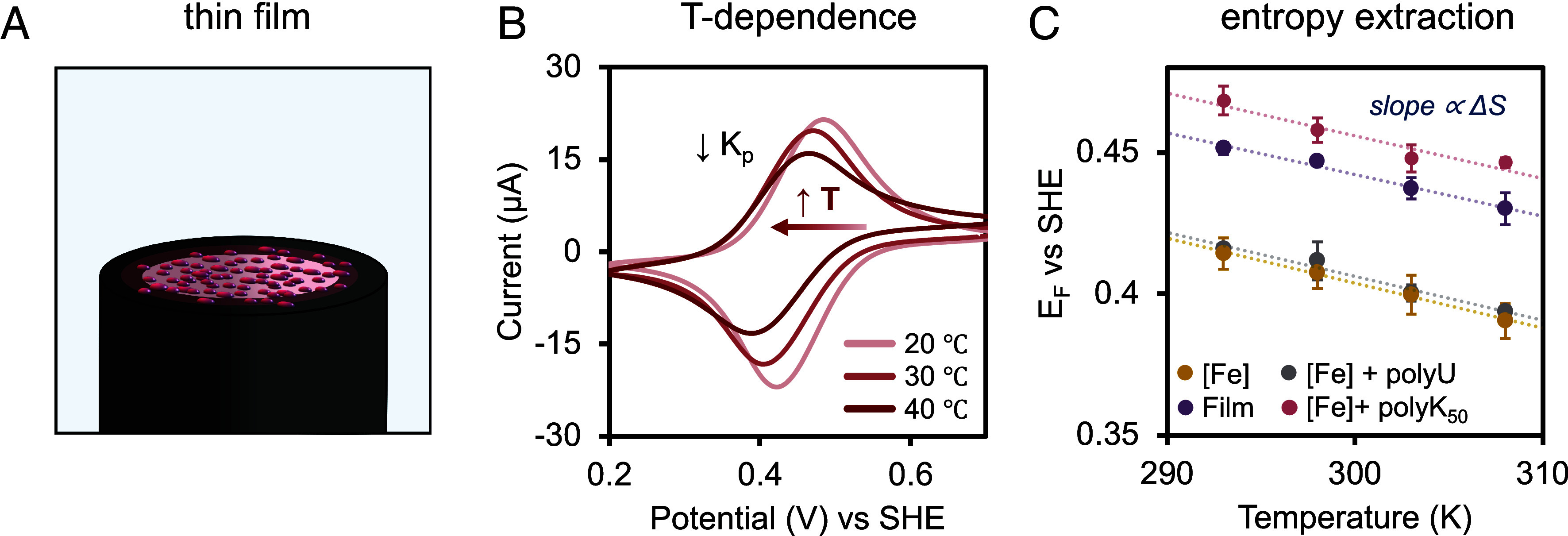
Method for thermodynamic investigation of electrochemical redox probes within thin films. (*A*) Schematic of a thin film electrode generated by drop casting LLPS droplets composed exclusively of polyK_50_ and polyU in water onto platinum working electrodes. (*B*) Temperature-dependent cyclic voltammograms of the thin film system with 1 mM ferrocyanide in 40 mM KCl electrolyte. A salt bridge was used to reference vs SCE at 20 °C (60 mV/s). (*C*) Plotted changes in the ferri/ferrocyanide, [Fe], formal potential with respect to temperature under varying conditions.

As the gradual precipitation of droplets onto the microelectrode surface only partially revealed the electrochemical behavior within the LLPS phase, we subsequently employed drop-casting of conformal coacervate thin films to enable more controlled and comprehensive electrochemical analysis. (Fig. 3*A* and *SI Appendix*, Fig. S10, see *SI Appendix* thin film deposition). All electrochemistry utilized a working solution of 40 mM KCl with 1 mM ferrocyanide, which partitions into the film within 3 min immersed in solution (see SI Electrochemistry). This technique is analogous to previously reported voltammetric experiments within coacervate dense phases and layer-by-layer films. (40–42) We observe a shift in formal potential with the films compared to in solution, and by performing scan rate dependence, the peak current increases linearly with scan rate, indicating that ferrocyanide is surface bound within the coacervate phase (*SI Appendix*, Fig. S11) (37). By changing the temperature of the system, we observe clear reductive shifts in E_f_ for both the thin film and bare electrode (Fig. 3*B* and *SI Appendix*, Fig. S12), indicating ferri/ferrocyanide abides by Eq. **3** in solution and in the film. As shown in Eq. **2**, a negative ∆S leads to an increase in ∆G with temperature, which in turn lowers E_f_ due to their inverse relationship (Eq. **1**). Note, the decrease in peak intensity with temperature is attributed to a 14% decreased partitioning of ferrocyanide rather than removal of the film upon exposure to higher temperatures (*SI Appendix*, Fig. S13). Conversely, ferricyanide demonstrates a 30% increase in partitioning at higher temperatures (*SI Appendix*, Fig. S14). The change in formal potential with respect to temperature reveals a reaction entropy of −121 ± 17 J/mol K within the film, which is 39 J/mol K more positive than that of the K_3/4_Fe(CN)_6_ in 40 mM KCl control (Fig. 3*C* and Table 1). This value suggests a more structured environment inside the coacervate phase. Since a ∆S of −133 J/mol K is obtained with polyK_50_ and ferro/ferricyanide, it is clear that polyK_50_ structures the majority of the solvent around the redox probe, with an additional −12 J/mol K provided by coacervation with PolyU. Together, polyK_50_ and polyU create a synergistic effect in the coacervate contributing to ferri/ferrocyanide’s altered reaction thermodynamics in addition to the polycation structuring. This additional solvent structuring is likely due to enhanced polymer–water interactions or polymer hydration which forms hydrogen bonds that are inaccessible in bulk solutions or concentrated solutions of polyelectrolytes that do not undergo LLPS (43, 44). We expect the decrease in reaction entropy in the presence of polyU may be due to the phosphate groups binding to the electrode surface and changing the local microenvironment. Reaction enthalpy within the film is −79.2 kJ/mol, which is 8 kJ/mol less exothermic than when compared to [Fe^II/III^] in KCl solution. This suggests that the HBN around the redox probe is strengthened within the film compared to when ferri/ferrocyanide is in the bulk. Interestingly, when a single polymer is in solution with the redox probe, regardless of charge, the redox reaction has an enthalpy of −83 kJ/mol, confirming that polymer charge has minimal influence on the reaction enthalpy. Together, these results follow intuitions whereby partitioning of a polar small molecule into a dense phase makes ET more energetically favorable due to the microenvironment stabilization of the charged species.

**Table 1.** Thermodynamic parameters extracted with ferri/ferrocyanide

Sample	E_f_ (V vs SHE)	∆G (kJ/mol)	∆H (kJ/mol)	∆S (J/mol K)
K_4/3_Fe^II/III^(CN)_6_	0.408 ± 0.002	−39.4 ± 0.2	−87.0 ± 5.2	−160 ± 17*
[Fe^II/III^] + polyU	0.412 ± 0.003	−39.8 ± 0.3	−83.6 ± 2.0	−147 ± 7*
[Fe^II/III^] + polyK_50_	0.458 ± 0.002	−44.2 ± 0.2	−83.8 ± 4.0	−133 ± 14
[Fe^II/III^] + film	0.447 ± 0.001	−43.1 ± 0.1	−79.2 ± 2.6	−121 ± 9

All measurements were done with 1 mM [Fe^II/III^] in 40 mM KCl with 1 mg/mL used for individual polymer samples and films. Differences between ∆S [Fe^II/III^] + film and other samples were tested for statistical significance using Student’s *t* test. *P < 0.05 of *t* values when compared with [Fe^II/III^] + film.

Tabulating all of the values extracted enables a broader perspective on the roles of each coacervate component. In the alkali metal series shown in *SI Appendix*, Table S1, increasingly structure-making ions decreases the exergonicity of the reaction. In contrast, we observe an increase in the reaction exergonicity with coacervate films, despite the reaction entropy suggesting a structured HBN microenvironment. We attribute this increase in exergonicity to the high ionic strength within the droplet, as we observe the same trend with increasing potassium concentration; −39.4 ± 0.2 kJ/mol in 40 mM KCl (Table 1) compared to Yang-Shao Horn’s data in 0.6 M KCl, −46.2 ± 0.7 kJ/mol (*SI Appendix*, Table S1). These findings highlight the dominant influence of ionic strength on redox reaction thermodynamics within coacervate microenvironments, even in the presence of structure-making ions. We expect that the similarly negative ∆G with polyK_50_ is due to the excess charge around the redox probe, making the local charge concentration sufficiently large, mimicking a high-salt concentration environment. Since the polyU control has a similar ∆G to 40 mM KCl and does not complex with ferri/ferrocyanide, this suggests that the presence of the negative polyelectrolyte species does not alter the reaction energy.

**Fig. 4.**
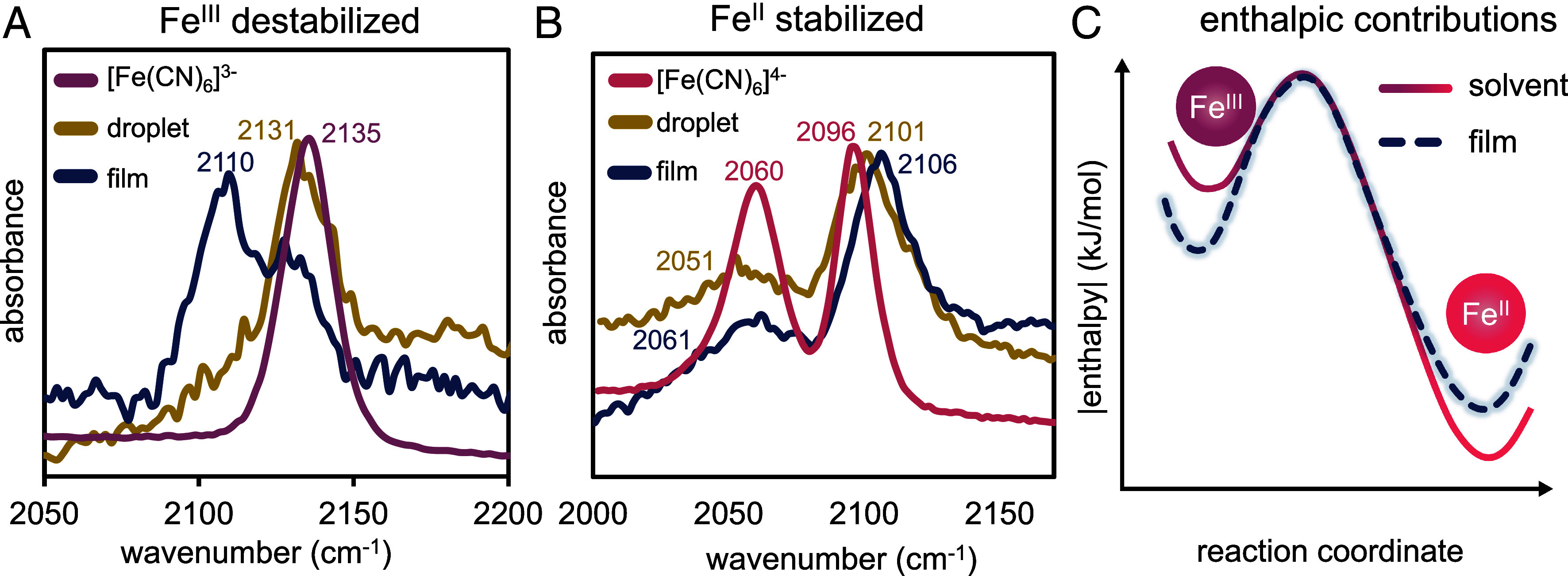
Raman spectroscopy reveals enhanced solvent structuring around Fe(III) and reduced structuring around Fe(II) within the coacervate phase. (*A*) Ferricyanide in solution (purple) has a singlet located at around 2,135 cm^−1^ which experiences a blue shift inside the droplet (yellow) toward 2,131 cm^−1^, and additional shift in the film to 2,110 cm^−1^ (blue). Peak magnitudes are altered to exemplify peak shifts. (*B*) Ferrocyanide in solution (pink) has two peaks located at 2,060 and 2,096 cm^−1^ that, respectively, correspond to the E_g_ and A_1g_ vibrational modes. Once coacervates crash out onto the coverslip (yellow), they demonstrate a nonconcerted shift toward 2,051 and 2,101 cm^−1^. When ferrocyanide is partitioned into a film (blue), it shows 2,060 and 2,106 cm^−1^. (*C*) Reaction coordinate diagram describing the absolute value of the enthalpy. We schematically describe a decrease in reaction exothermicity of Fe(III) and an increase in reaction exothermicity with Fe(III) in the film (purple) versus in solution (pink).

To further understand the local environment within the coacervate phase, Raman spectroscopy, in the form of optical-photothermal infrared and Raman spectroscopy (O-PTIR), was performed. This technique was chosen because of its high lateral spatial resolution (ca. 500 nm) and ability to resolve the changes in CN stretching frequencies of the ferri- and ferrocyanide molecules due to changes in their secondary coordination sphere. Control measurements were performed in solution, while measurements of the coacervate phase were made on droplets and films adhered to the bottom of a glass microscope slide. While potassium ferrocyanide, K_4_Fe(CN)_6_, in 40 mM KCl exhibits two distinct CN stretching frequencies at 2,060 and 2,096 cm^−1^, the two features converge in the oxidized potassium ferricyanide, K_3_Fe(CN)_6_, state at 2,135 cm^−1^. The ferricyanide peak position red shifts 4 cm^−1^ in the droplets and 25 cm^−1^ in the film (Fig. 4*A*). Ferricyanide’s red-shifted value is consistent with the coacervate phase and film having a strongly hydrogen-bonded water framework solvating ferricyanide’s charge. It has been shown with alkali metals that increasing “structure-making” character results in the red shifting of the CN stretching frequencies(45, 46). In the presence of PolyK_50_ and PolyU, Fe(III) shifted up to 2 cm^−1,^ suggesting solvation changes are not a result of individual polymer interactions with ferricyanide (*SI Appendix*, Fig. S15*A*). However, the ferrocyanide A_1g_ mode exhibits a blue shift of 5 and 10 cm^−1^ for the droplet and film, respectively, and the E_g_ vibrational mode shows a 9 cm^−1^ red shift in the droplet phase and an insignificant shift in the film (Fig. 4*B*). This unexpected deviation points to a weak or asymmetric solvation shell around Fe(II), potentially due to nonideal spacing along the backbone minimizing electrostatic interactions between lysine’s amine and the iron center—an observation that is further supported by ferrocyanide’s 4 cm^−1^ shift with polyK_50_ (*SI Appendix*, Fig. S15*B*). Additionally, it may also suggest that the structured environment creates and stabilizes strong dipole-generating vibrational modes, due to the presence of dipole–dipole interactions between polyelectrolytes that are essential to phase separation (47). Together, we identify the reason for the decrease in reaction exothermicity within the coacervates as both ferricyanide stabilization and ferrocyanide destabilization (Fig. 4*C*). Further, we note that the film exacerbates shifts relative to droplets, indicating that while coacervate films are not perfect models to study in-solution coacervate microenvironments, they act as a good proxy to study coacervate trends. These results are intuitively consistent with the oxidation of the Fe(II) state occurring at more positive potentials, or, in other words, the internal environment of the coacervate being more oxidizing.

## Conclusion

In this work, we quantify enthalpic and entropic contributions toward single ET thermodynamics within a coacervate phase utilizing temperature-dependent electrochemistry and Raman spectroscopy. By measuring the formal potential over a narrow temperature window, we quantify redox reaction thermodynamics inside and outside coacervates. We then utilize Raman to qualitatively validate our electrochemical results by studying CN stretching frequency shifts in solution, droplets, and films. Relating these shifts to the solvation structure around the probe, we reveal water structure and polymer electrostatics as key factors that influence the thermodynamics of the model ferri/ferrocyanide redox couple confined within the coacervate phase. Both these factors, respectively, increase ∆∆S and ∆∆H by 39 J/mol K and 7.8 kJ/mol, which is consistent with the water being highly structured within the coacervate phase. This difference results in a ∆∆G = −3.8 kJ/mol for a single ET between ferricyanide and ferrocyanide. Raman spectroscopy reveals tight solvation of ferricyanide and the loose solvation of ferrocyanide, likely due to the structural rigidity of polyK_50_, which is commensurate with a decrease in reaction exothermicity within the coacervate phase. Selectively stabilizing the oxidized ferricyanide state is also consistent with the internal environment of similar coacervates being reported as “oxidizing” in nature, as the barrier for the oxidation of ferrocyanide is reduced within the coacervate phase. The spectroscopy also demonstrates these interactions are more pronounced in films than in droplets, likely due to film’s lower water content. Together, these results suggest that films are acceptable proxies for coacervate droplets but may exaggerate any effects observed in solution. Overall, the electrochemical and spectroscopic insights of this work provide a platform for future studies of redox reactivity within coacervate phases, whether in the droplet phase or in a thin film state. Finally, we argue that describing coacervates as merely promiscuous catalysts is potentially misleading, as the reaction Gibbs energies of both reactants and products are altered relative to the bulk, leading to changes in the equilibrium constant.

## Materials and Methods

### Materials.

Poly-(L-lysine hydrochloride) (PolyK_50_; degree of polymerization N = 50, and Mn = 8.2 kDa) was purchased from Alamanda Polymers. Polyuridylic acid potassium salt (polyU, polydisperse) was purchased from Sigma Aldrich. Both polypeptides were used directly without further purification. Potassium ferricyanide [K_3_(Fe(CN)_6_), Sigma Aldrich, ACS Reagent, ≥99.9%] and potassium hexacyanoferrate [K_4_(Fe(CN)_6_), Sigma Aldrich, ACS Reagent, ≥ 99.9%], and potassium chloride (KCl, Sigma Aldrich, ≥99%) were used as received. DI water was sourced from a MilliQ purification system (resistivity 18.6 MΩ cm at 25 °C). For the sake of clarity, all polymer concentrations are reported in terms of polymer units instead of their monomeric concentrations.

### Turbidity Measurements.

Time- and temperature-dependent measurements were performed on a TECAN M200 Infinite Pro plate reader. Then, 100 µL of solution was used with half-area 96-well plates with transparent lids to limit evaporation. Orbital mixing was performed between the measurements taken every 2.5 min, where the absorbance at 600 nm was taken and averaged across at four locations within each well. No spatial differences within the cells were observed. Turbidity was calculated using the following equation:

$$T=100-\left(10^{-\mathrm{Abs}\;600\;{\mathrm{nm}}^{*}}100\right)$$

Critical salt concentration curve iron titration curves were performed using a Shimadzu UV-1800 spectrophotometer with 10.0 mm pathlength cuvettes (Firefly Scientific).

### Dynamic Light Scattering.

Dynamic light scattering (DLS) was performed with a Malvern Zetasizer Nano ZS. Measurements were conducted at the indicated temperature with 150 μL volumes. All samples were probed with a 632.8-nm HeNe gas laser with a beam diameter of 0.63 mm (1/e^2^) and detected by an avalanche photodiode (quantum efficiency >50% at 633 nm) in a backscattering configuration at 173°.

### Thin Film Deposition.

A solution of 1 mg/mL polyK_50_ and 1 mg/mL polyU in water was prepared by mixing and equilibration for 5 min. Then, 15 µL were then drop casted onto a 2 mm diameter polished platinum electrode and left to dry at 60° C for an hour in the oven.

### Electrochemistry.

All electrochemical voltammetry experiments were performed on a Biologic VMP3 potentiostat. Cyclic voltammetry experiments were carried out with a 2 mm diameter platinum working electrode (CH Instruments), a saturated calomel electrode (SCE) as reference electrode (CHI150, CH Instruments), and a Pt wire counter electrode (CH Instruments) in a conventional three-electrode cell. Platinum working electrodes were polished for 2 min on Buehler 8 in microcloth polishing pads using 3, 1, 0.25, 0.05 µm diamond suspensions (Buehler). All voltammograms are presented in the IUPAC convention (reductive currents are negative). All electrochemical experiments were performed at the indicated temperature in 40 mM KCl with 1 mM ferri/ferrocyanide. Electrochemical impedance was performed at open circuit potential to determine the solution resistance at each condition to calculate the IR drop. An IKA stirring hot plate was used to set the temperature using an aluminum heating mantle. Homemade salt bridges with sat. KCl were used for elevated temperature measurements using CoralPor tips from BASi. Diffusion coefficients were calculated using the Randles Sevcik Equation using known concentrations of potassium ferro/ferricyanide and by varying scan rate from 10 mV/s to 100 mV/s.

### Optical Photothermal Infrared and Raman Spectroscopy (OPTIR).

Measurements were conducted on a Photothermal Spectroscopy Corp O-PTIR with 10× and 50× magnifying lenses. Sample solutions were made and analyzed either in a thin glass cuvette (1 mm path length) or microscope slide with vacuum grease and a cover slip used to prevent the drying of the sample. Measurements performed at 10× magnification were taken using 5 s integration time with 5 averages per spectrum at 3.5% laser intensity, with an open pinhole and 600 resolution. Measurements performed at 50× magnification were taken using 5 s integration time with 5 averages per spectrum at 1.25% laser intensity, with an open pinhole and 1200 resolution. For film samples, 15 s integration time with 5 averages per spectrum and 6–10% laser intensity was required. Note only Raman spectra were collected as the instrument did not have an IR detector for the 1842–2650 cm^–1^ region of interest.

### Partitioning Determined by UV-Vis Spectroscopy.

A solution of 1 mg/mL polyK_50_, 1 mg/mL polyU and 1 mM K_3_[Fe(CN)_6_] or K_4_[Fe(CN)_6_] in 40 mM KCl was prepared by mixing and equilibration for 10 min at the indicated temperature. Once stable, the coacervated solution was centrifuged at 6000 rpm for 20 min at the indicated temperature. The supernatant was extracted and analyzed using the Shimadzu UV Vis Spectrometer UV-1800. All UV-Vis spectra were acquired using medium speed and 1 nm sampling intervals using Brand UV-cuvettes micro. Absorbance peaks at 320 and 425 nm were used to calculate concentrations of Fe(II) and Fe(III), respectively. An extinction coefficient of 651 mol/L cm was used for K_4_[Fe(CN)_6_] and 1020 mol/L cm for K_3_[Fe(CN)_6_] as determined by a concentration calibration.

## Supplementary Material

Appendix 01 (PDF)

## Data Availability

All study data are included in the article and/or *SI Appendix*.
