# Supplementary material for: Quantification of redox thermodynamics shifts within coacervates
Source: Proc Natl Acad Sci U S A. 2025 Nov 14;122(46):e2521526122. doi: 10.1073/pnas.2521526122 (PMC12646308; doi:10.1073/pnas.2521526122)
Supplement: Supplementary file 1 — Appendix 01 (PDF) [file pnas.2521526122.sapp.pdf]

Supporting Information: Quantification of Redox Thermodynamics Shifts Within Coacervates

Gala Rodriguez,‡ Nicholas B. Watkins,‡ Xagros Faraji, Elizabeth Lee, Lior Sepunaru*
Department of Chemistry and Biochemistry, University of California at Santa Barbara, Santa Barbara, CA 93106, United States
*email: sepunaru@ucsb.edu

Page	
2	Table S1: Ferri/Ferrocyanide thermodynamic data with various alkali metals
2	Figure S1: Identifying model coacervate system with DLS
3	Figure S2: LLPS time-dependent DLS under variable temperatures
3	Figure S3: LLPS optical image
4	Figure S4: Temperature-dependent turbidity
4	Figure S5: Time-dependent turbidity
5	Figure S6: Formal potential shifts as a function of polymer concentrations
5	Figure S7: Setup for inverted microelectrode droplet cell
6	Figure S8: First twenty scans of microelectrode data
6	Figure S9: Microelectrode with and without droplets at fast scan rates
7	Figure S10: Optical image of a thin-film drop cast onto Pt electrode
7	Figure S11: Scan rate dependence and analysis in thin films
8	Figure S12: Temperature-dependent 0.2 mM ferro/ferricyanide cyclic voltammograms
8	Figure S13: Film withstands high temperatures
9	Figure S14: Temperature-dependent partition coefficients
9	Figure S15: Raman control experiments

Table S1: Tabulated thermodynamic information for the ferri/ferrocyanide reaction at a platinum electrode in 0.6 M MCl electrolytes, where M= Li^+, Na^+, K^+, Rb^+,and Cs^+. ΔH is calculated at 20 ºC using the free energy equation given experimental ΔG and ΔS values. Data obtained from Ref. [29] from the main text.

Sample	E_0 (20 ºC) (V vs SHE)	ΔG (kJ/mol)	ΔH (kJ/mol)	ΔS (J/mol K)
$Li_{4/3}Fe^{II/III}(CN)_6$	0.468 ± 6	-45.2 ± 0.5	-86.4 ± 0.8	-141 ± 2
$Na_{4/3}Fe^{II/III}(CN)_6$	0.469 ± 5	-45.3 ± 0.4	-88.5 ± 0.9	-147 ± 3
$K_{4/3}Fe^{II/III}(CN)_6$	0.479 ± 7	-46.2 ± 0 .7	-89.7 ± 1.1	-149 ± 3
$Rb_{4/3}Fe^{II/III}(CN)_6$	0.491 ± 7	-47.3 ± 0.7	-91.8 ± 1.3	-152 ± 4
$Cs_{4/3}Fe^{II/III}(CN)_6$	0.498 ± 5	-48.1 ± 0.5	-96.9 ± 1.2	-167 ± 4

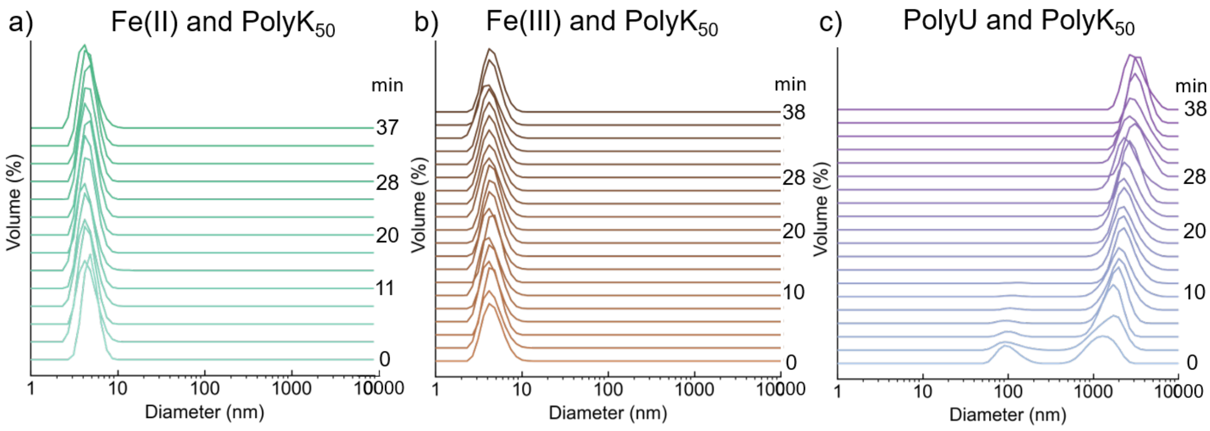

Figure S1: **PolyU participation in coacervation.** a) 1 mM Ferrocyanide with 1 mg/mL $PolyK_{50}$ in 40 mM KCl b) 1 mM Ferricyanide with 1 mg/mL $PolyK_{50}$ in 40 mM KC c) 1 mg/mL PolyU with 1 mg/mL $PolyK_{50}$ in 40 mM KCl. All DLS readings are taken at room temperature.

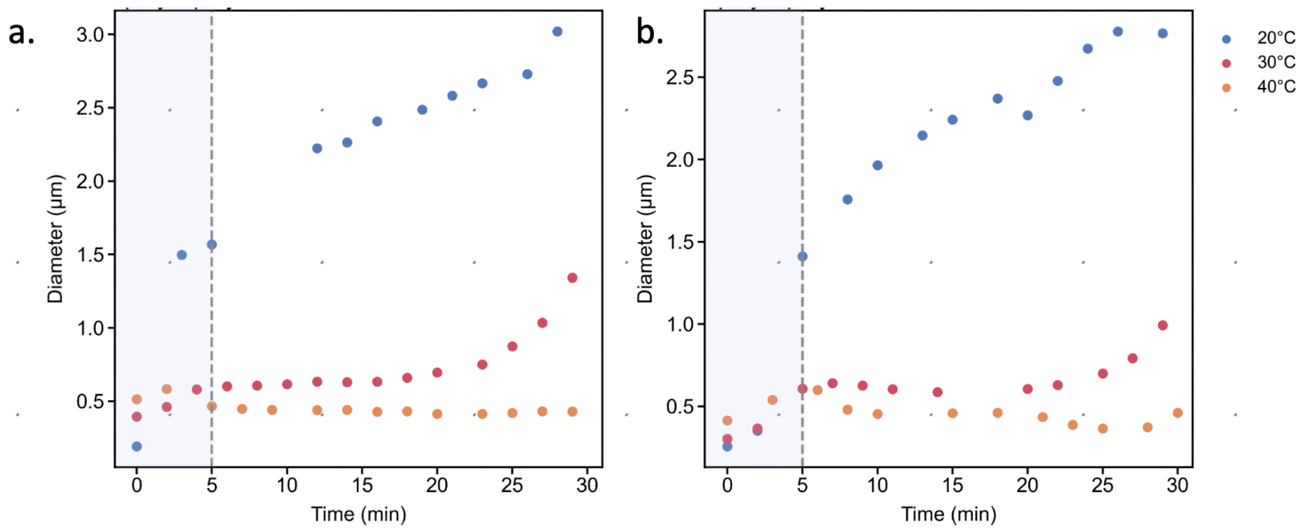

Figure S2: **Time-dependent DLS measurements under variable temperatures.** a) polyU + polyK$_{50}$ LLPS + Fe(II) and b) polyU + polyK$_{50}$ + Fe(III) LLPS at various temperatures. All measurements were taken in 40 mM KCl with 1 mg/mL polymer concentrations. 1 mM Fe(II) or Fe(III) was added to the polyU and polyK$_{50}$ mixture after 5 minutes of equilibration (dashed line).

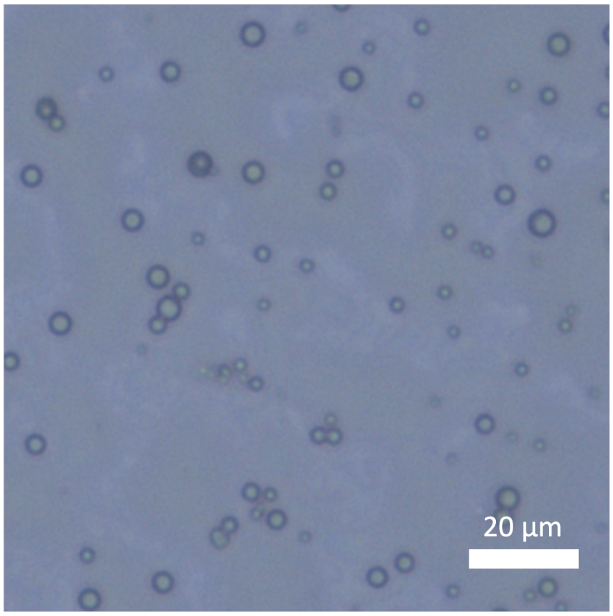

Figure S3: **Optical image of 1 mg/mL polyU + polyK$_{50}$ + 1 mM Fe(II) LLPS in 40 mM KCl.**

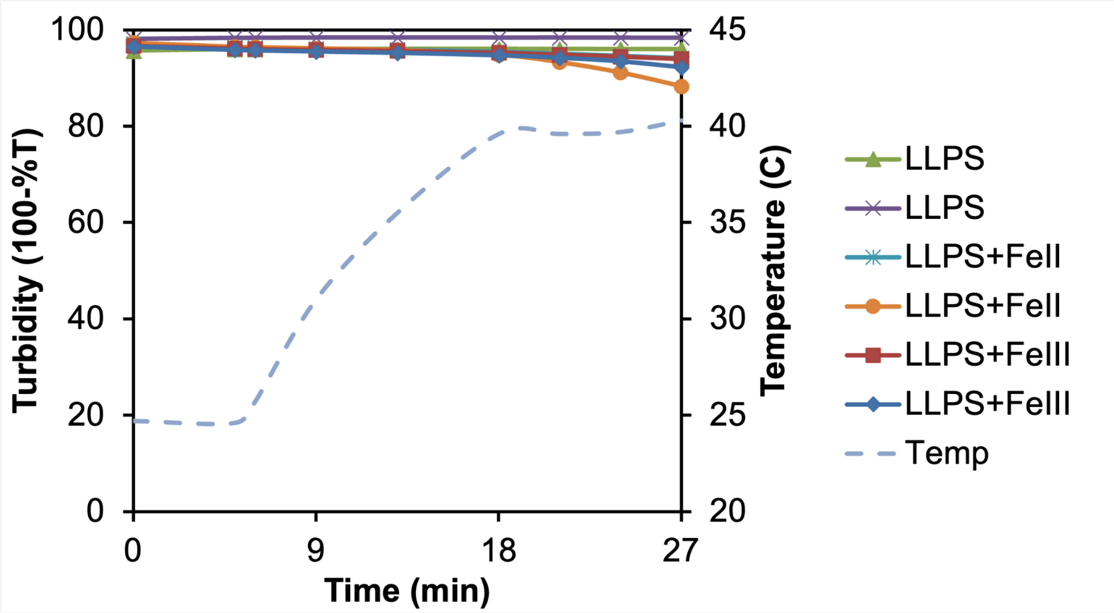

Figure S4: **Temperature-dependent turbidity measurements.** a) PolyU + polyK$_{50}$ LLPS b) polyU + polyK$_{50}$ + Fe(II) LLPS c) polyU + polyK$_{50}$ + Fe(III) LLPS

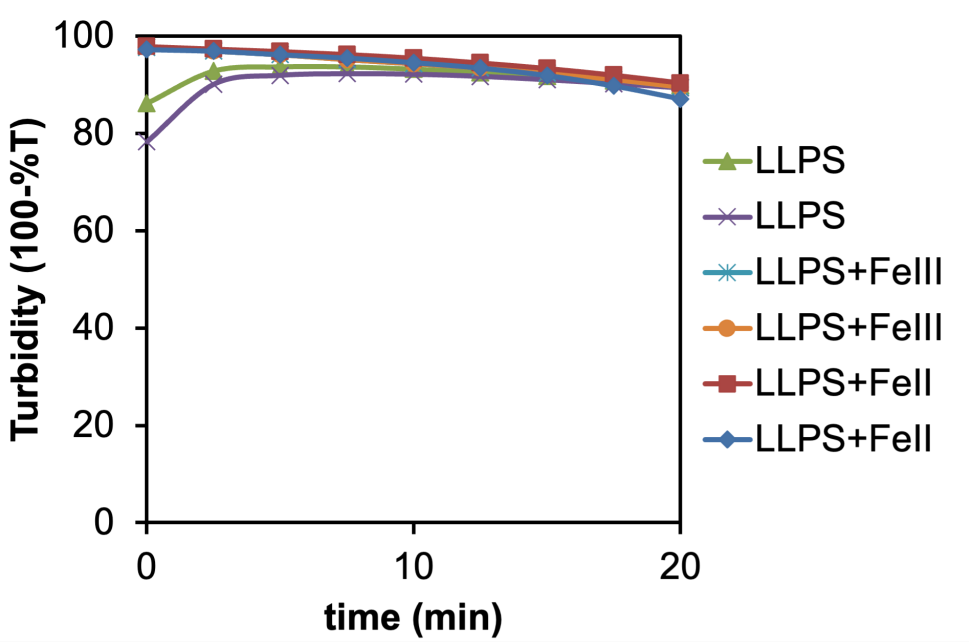

Figure S5: **Time-dependent turbidity measurements.** PolyU + polyK$_{50}$ LLPS with and without each iron species with 1 mg/mL PolyK$_{50}$ and 1 mg/mL polyU, and if an iron complex was present, it was 1 mM. Performed at 20 ºC.

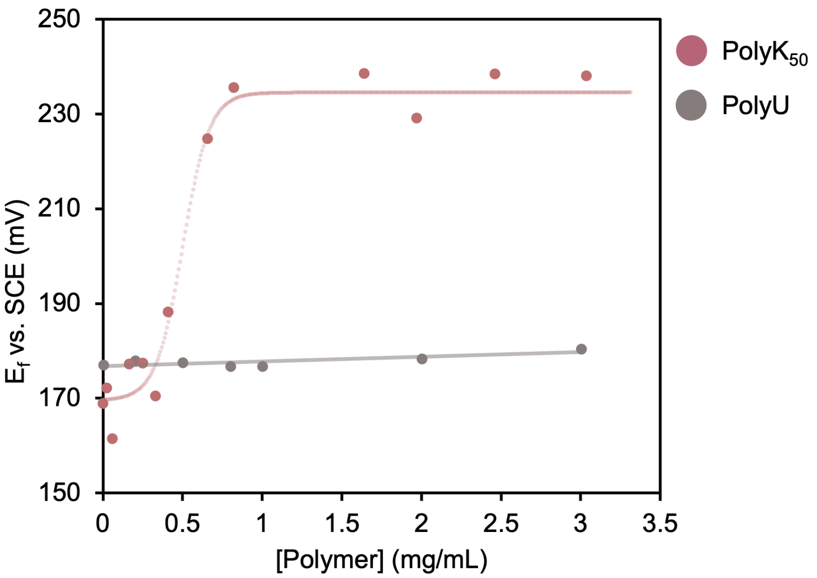

Figure S6. Formal potential shifts as a function of PolyK$_{50}$ concentration but independent of PolyU concentration. Solutions of the respective polymer concentrations were made in 40 mM KCl buffer with 1 mM Ferrocyanide. The ferri/ferro redox couple's formal potential increases by 60 mV when the solution is saturated polyK$_{50}$ but remains unchanged when saturated with PolyU.

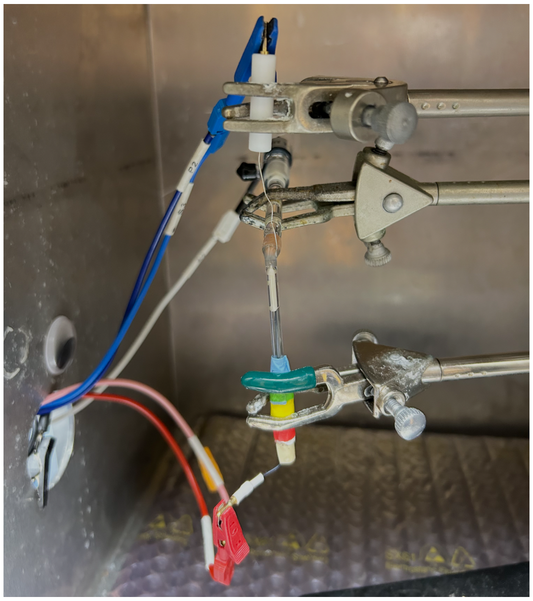

Figure S7: Setup for inverted microelectrode droplet cell. Clamps are used to fix a platinum microelectrode, the platinum wire counter electrode, and the reference electrode. 25 µL of solution is used to immerse all three components.

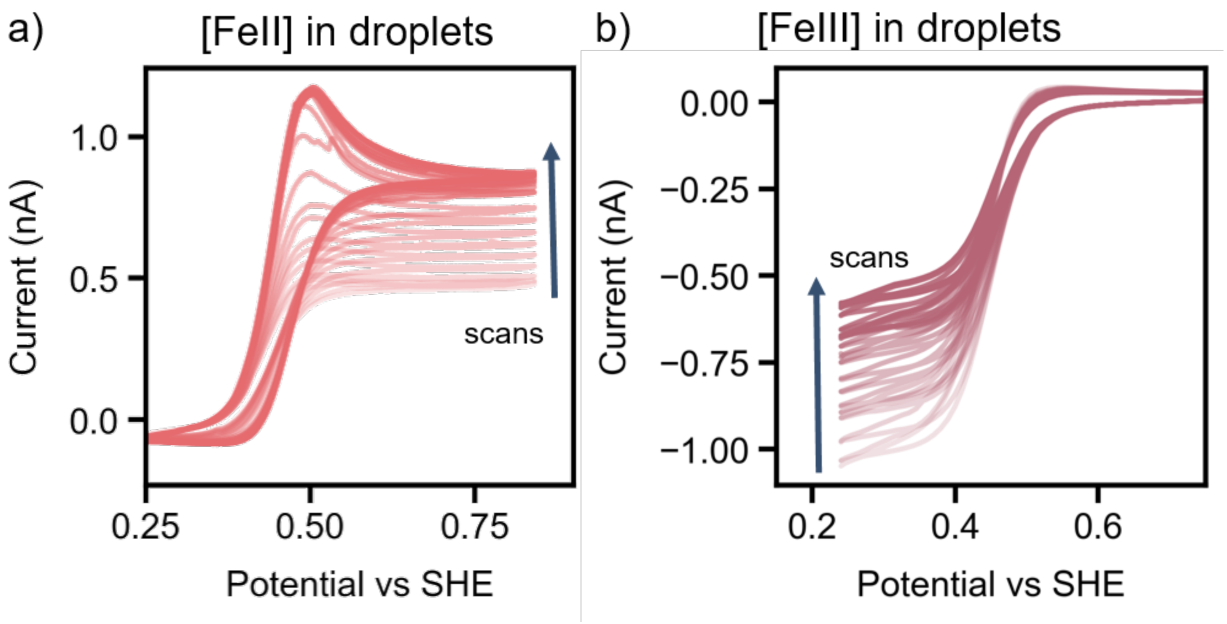

Figure S8: First twenty scans of microelectrode data. a) 1 mM ferrocyanide with 1 mg/mL of polyK$_{50}$ and polyU. b) 1 mM ferricyanide with 1 mg/mL of polyK$_{50}$ and polyU. Both figures utilize a 20 mV/s scan rate, 40 mM KCl buffer with a 10 µm Pt electrode and Pt wire counter electrode.

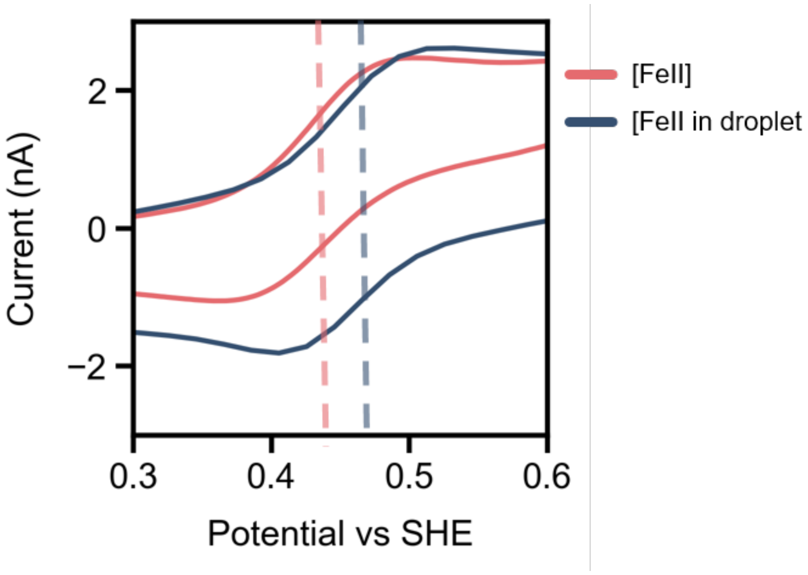

Figure S9: Microelectrode with and without droplets at fast scan rates. a) 1 mM ferrocyanide (pink) with 1 mg/mL of polyK$_{50}$ and polyU (blue). 10 V/s scan rate, 40 mM KCl buffer with a 10 µm Pt electrode and Pt wire counter electrode.

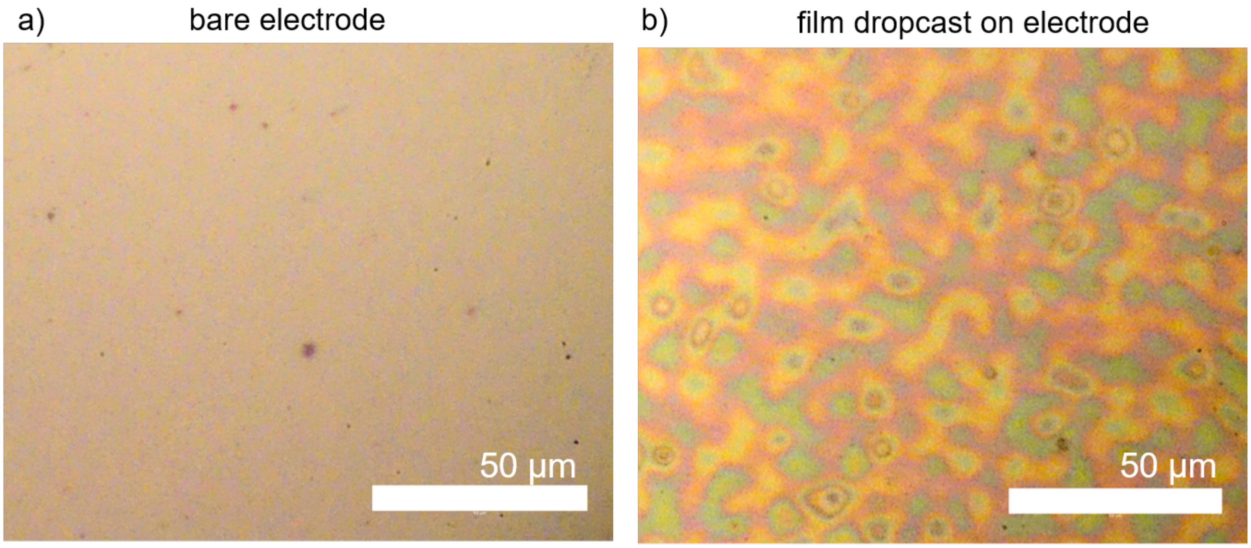

Figure S10. Optical image of a) bare Pt electrode and b) Pt electrode with a film. The surface has an inhomogeneous color, we clearly see the majority of the electrode is coated with when compared to the bare electrode. Variation in color is due to changes in film's refractive index suggesting some parts have a higher concentration of polymer (yellow) from coacervates, and others have a lower polymer concentration (blue).

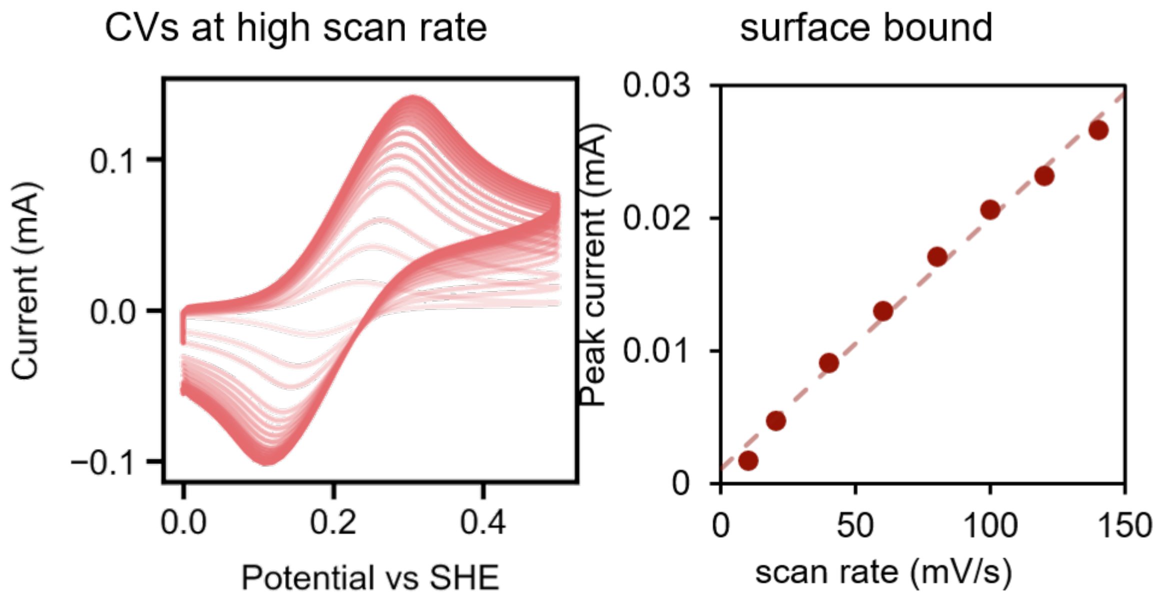

Figure S11. Ferrocyanide voltammetry within coacervate films exhibits surface-bound behavior. a) Scan-rate dependent CVs of a LLPS film Pt covered electrode with a 1 mM ferrocyanide in 40 mM KCl solution. Scan rates are from 10 mV/s to 140 mV/s. b) The slope of peak current versus scan rate has an R = 0.992 suggesting the probe behaves similarly to surface bound species within the film.

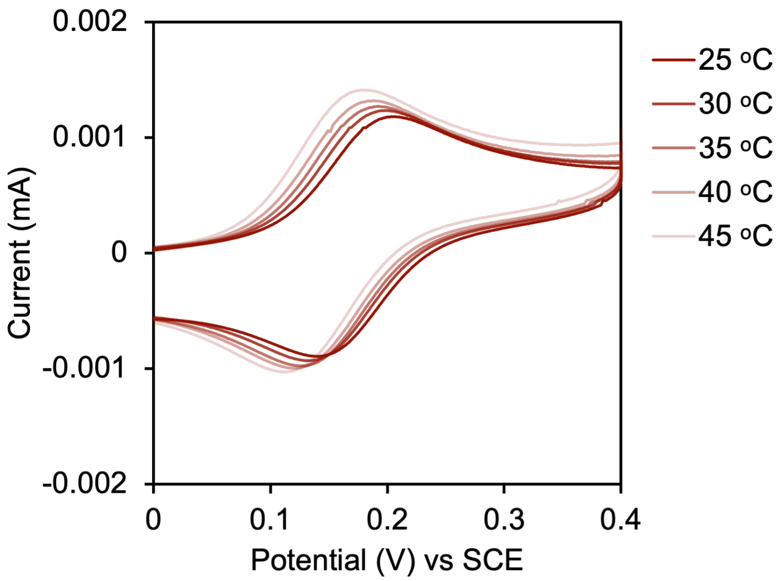

Figure S12: **Temperature-dependent 0.2 mM ferro/ferricyanide cyclic voltammograms.** Performed in 40 mM KCl taken with a Pt electrode at 60 mV/s.

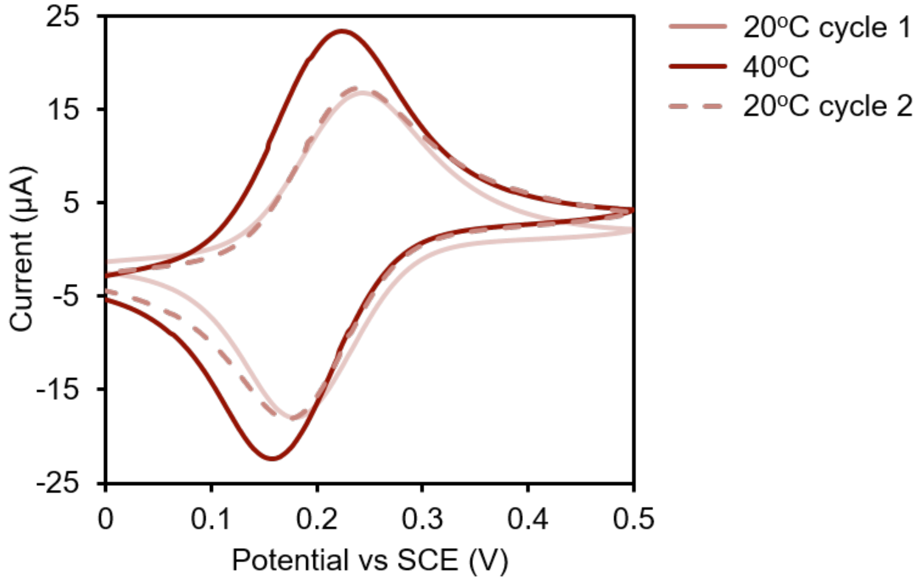

Figure S13. Film withstands high temperatures. Cyclic voltammograms were taken of 1 mM ferrocyanide in 40 mM KCl with a film coated Pt electrode at 20°C, 40°C, and back down to 20°C. Between temperatures the electrode was taken out of solution until the next CV was taken.

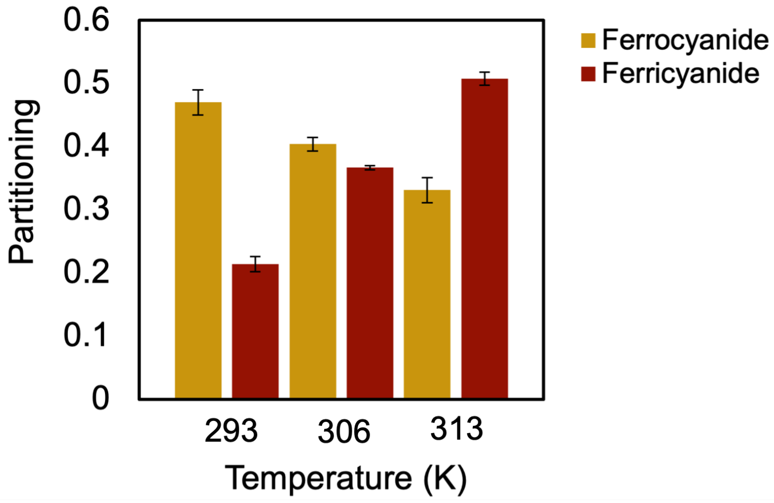

Figure S14: **Temperature-dependent partition coefficients for 1 mM Fe(II) and Fe(III) in 1 mg/mL polyU + polyK$_{50}$ LLPS and 40 mM KCl.** The solutions were left to equilibrate for 10 minutes then centrifuged for 20 minutes at the relevant temperatures. Following centrifugation the supernatant was analyzed with UV-Vis at 320 nm and 425 nm for Fe(II) and Fe(III) respectively.

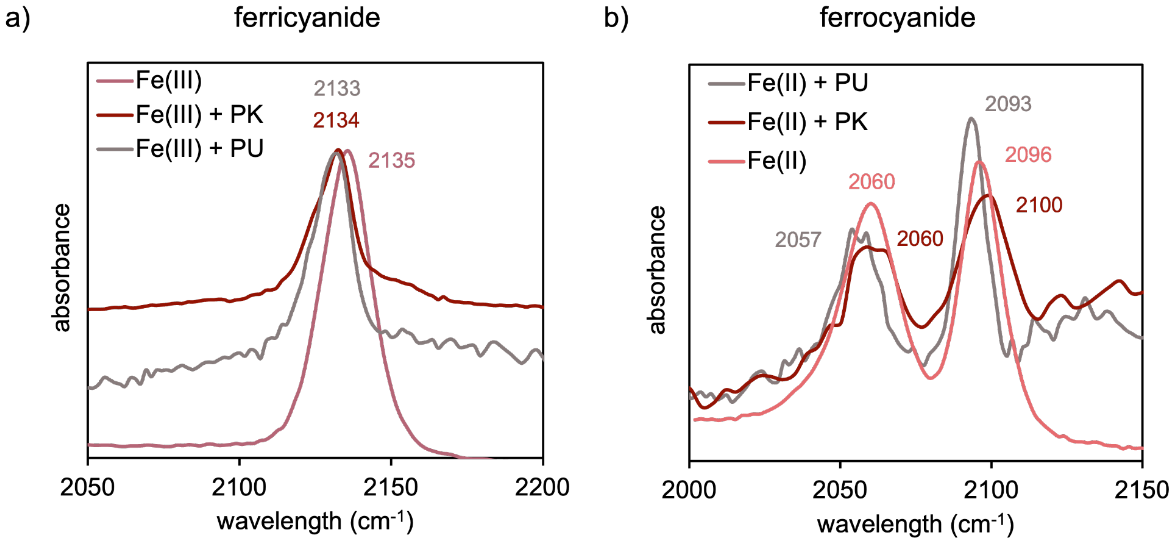

Figure S15: **Raman control experiments.** Raman characterization of 10 mM ferri/ferrocyanide with 10 mg/mL coacervate components in 40 mM KCl. Peak heights are not representative of concentrations.
